# Comparison of the Efficacy of Pre-procedural Rinses Using 0.2% Chlorhexidine Gluconate Versus a Stabilized Chlorine Dioxide Mouthwash in Patients With Chronic Periodontitis: A Clinico-Microbiological Study

**DOI:** 10.7759/cureus.110231

**Published:** 2026-06-04

**Authors:** Charu Kalra, Shruti Tandon, Arundeep K Lamba, Sonal Saxena, Farrukh Faraz

**Affiliations:** 1 Periodontics and Oral Implantology, Maulana Azad Institute of Dental Sciences, New Delhi, IND; 2 Dentistry, Sub-District (SDH) Hospital, Lunkaransar, IND; 3 Microbiology, Maulana Azad Medical College, New Delhi, IND

**Keywords:** 0.2% chlorhexidine gluconate, aerosol contamination, colony-forming units, periodontitis, pre-procedural mouth rinse, stabilized chlorine dioxide, vas score

## Abstract

Background: Aerosols produced during dental procedures carry a high microbial load, increasing the risk of cross-infection between patients and dental professionals. The use of pre-procedural mouth rinses is a simple, cost-effective, and practical measure to reduce microbial contamination in aerosols generated during dental treatment.

Aim: This study aimed to assess and compare the effectiveness of 0.2% chlorhexidine gluconate (CHX) and 0.1% stabilized chlorine dioxide (ClO₂) when used as pre-procedural rinses in patients with chronic periodontitis.

Materials and methods: A total of 48 systemically healthy individuals with chronic periodontitis were randomly allocated to two groups: Group I (CHX) and Group II (ClO₂), with 24 participants in each group. Unstimulated saliva samples were obtained before and after rinsing. Microbial evaluation was done by quantifying aerobic and anaerobic colony-forming units (CFUs). Participants also recorded their perception of taste and odor using a Visual Analog Scale (VAS). Data were analyzed using paired and unpaired t-tests, with significance considered at p ≤ 0.05.

Results: CHX showed a significant reduction (81.96%) in aerobic culture, whereas ClO₂ showed no statistical significance in reduction (40.91%) in aerobic culture. Both rinses produced a significant decline in anaerobic CFU counts; Group I showed an 86.05% reduction, and Group II showed a 79.75% reduction in microbial load. ClO₂ was rated higher on the VAS scale, reflecting better taste and odor acceptability.

Conclusion: Both CHX and ClO₂ effectively lowered anaerobic bacterial counts. While CHX had a stronger effect against aerobic bacteria, ClO₂ offered greater patient acceptability, supporting its use as a viable alternative in routine dental practice.

## Introduction

Dental procedures involving ultrasonic scalers, air-water syringes, and high-speed handpieces generate aerosols - tiny droplets measuring ≤5 µm - that can remain airborne and travel considerable distances within the clinical environment [1]. While essential for instrument cooling and procedural efficiency, these aerosols pose a significant risk of airborne transmission of infectious agents because aerosols settle down slowly, can be inhaled deep into the lungs, and can drift far away with air currents [2]. Splatter is more than 50 micrometers in size, can be seen with the naked eye, and contaminates the immediate surface, the operator (face shield, hands, and chest), and the floor. Additionally, infection could be transmitted from the eyes or nose, or through direct contact [3]. Both often contain a mixture of saliva, blood, bacteria, and viruses, increasing the risk of cross-contamination among patients and dental healthcare personnel, especially during aerosol-generating procedures such as ultrasonic scaling and tooth extractions [4].

Environmental contamination is another concern, as aerosolized particles can settle on surrounding surfaces and contribute to indirect transmission [5]. The risk to dental professionals was underscored during the COVID-19 pandemic, when aerosol transmission of severe acute respiratory syndrome coronavirus 2 (SARS-CoV-2) became a serious occupational hazard [6].

To mitigate these risks, several infection control strategies have been recommended. One of the most effective measures is the use of pre-procedural mouthrinses containing antimicrobial agents such as chlorhexidine, stabilized chlorine dioxide (ClO₂), and povidone-iodine, which can significantly reduce the microbial load in the oral cavity and, consequently, in aerosols [7-9]. Chlorhexidine gluconate (CHX) is widely regarded as the gold standard due to its broad-spectrum antimicrobial activity, whereas stabilized ClO₂ acts through protein oxidation and disruption of microbial cell integrity. Povidone-iodine and essential oils are also used for their antiviral and antibacterial properties [8,10].

In addition, strict adherence to personal protective equipment protocols, including N95 or KN95 masks, face shields, gloves, and gowns, is essential to prevent inhalation of and contact with contaminated particles [11]. Environmental controls such as high-efficiency particulate air filters, increased air exchange rates, and surface disinfection with Environmental Protection Agency-approved agents further enhance safety within dental operatories [12]. Modifications in treatment, such as rubber dam isolation and the use of reduced aerosol-generating techniques, provide additional layers of protection.

The importance of these measures is further emphasized in periodontal care. Periodontal disease, a chronic inflammatory condition characterized by deep periodontal pockets, facilitates microbial access to the bloodstream, potentially leading to bacteremia [13]. Even routine oral hygiene practices or mastication can induce transient bacteremia in affected patients. In advanced cases, the resulting systemic inflammatory burden may contribute to cardiovascular disease, diabetes, and other systemic conditions [14].

Therefore, reducing the oral microbial load and controlling aerosol generation are critical not only for infection prevention in the dental operatory but also for safeguarding overall systemic health.

Commercially available mouthwashes containing povidone-iodine, chlorhexidine, cetylpyridinium chloride (CPC), zinc lactate, sodium fluoride, and stabilized ClO₂ claim to reduce bacterial counts. In this study, a comparison of the efficacy in reducing microbial load was conducted between 0.2% CHX and 0.1% stabilized ClO₂ mouthwash when used as a pre-procedural rinse.

## Materials and methods

The present study was a single-blind (the lab technician performing the culture analysis was blinded), randomized clinical (randomization was done using computer-generated random allocation software), and microbiological trial conducted from May 2023 to November 2024. It was approved by the Institutional Ethics Committee of Maulana Azad Institute of Dental Sciences (approval no.: 2023/449) and registered with the Clinical Trials Registry-India (CTRI) (CTRI/2023/05/053240 and REF/2023/05/067023), as it was a randomized trial. This study aimed to evaluate and compare the efficacy of 0.2% CHX and 0.1% stabilized ClO₂ in reducing the salivary microbial load (colony-forming unit (CFU) count) following a pre-procedural rinse.

Sample size

It was revealed from the literature survey [15] that the expected mean ± SD of the parameters at two intervals were 17.31 ± 6.91 and 24.71 ± 6.78, respectively. Based on a power analysis of this data using G*Power version 3.1 software (Heinrich Heine University Düsseldorf, Düsseldorf, Germany), a two-tailed test yielded an effect size of 1.081 and 95.0% power. Therefore, for an α error probability of 5% and a 95% confidence interval, the sample size was 24 per group, for a total of 48 across the two groups.

Means: difference between two independent means (two groups). Analysis: a priori: compute the required sample size. Input: Tail(s) = two. Effect size d = 1.0810323; α err prob = 0.05; power (1-β err prob) = 0.95; allocation ratio N2/N1 = 1; output: noncentrality; parameter δ = 3.7448057; critical t = 2.0128956; Df = 46; sample size group 1 = 24; sample size group 2 = 24; total sample size = 48.

Inclusion criteria

Subjects of either sex, aged between 30 and 50 years, diagnosed with chronic periodontitis (according to the newer classification (a) Generalized Periodontitis Stage II Grade B and (b) Generalized Periodontitis Stage III Grade B) and having a minimum of 20 natural teeth, with a probing depth of ≥5 mm, clinical attachment loss of ≥5 mm in at least 30% of sites, and bleeding on probing in at least 30% of teeth, were screened for the study and enrolled after providing informed consent.

Exclusion criteria

Subjects with orthodontic or prosthetic appliances, mouth ulcers, a history of recent tooth extraction or periodontal surgery within the past six months, those using regular mouthrinses, or those who had taken antibiotics within the last five days were excluded. Smokers, tobacco chewers, pregnant or lactating women, and individuals with a history of allergy to any mouthwash were also excluded.

Experimental design, randomization, and study procedure

**Figure 1:**
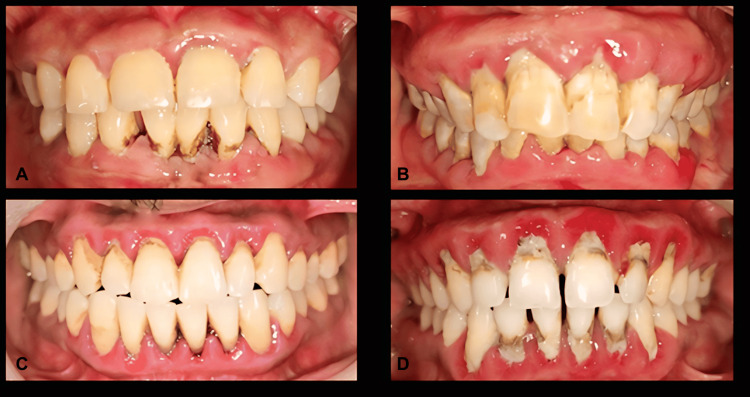
Patients with chronic periodontitis selected for the research (A-D) Probing depth, clinical attachment loss, and bleeding on probing were measured for each individual patient.

**Figure 2:**
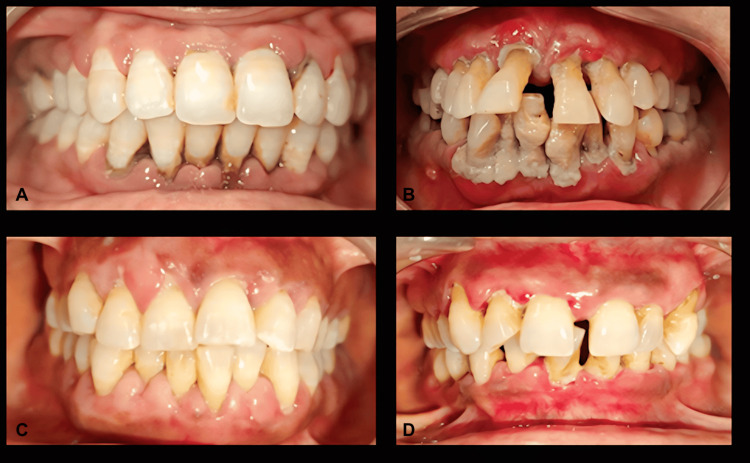
Patients with chronic periodontitis selected for the research (A-D) Probing depth, clinical attachment loss, and bleeding on probing were measured for each individual patient.

Patients attending the outpatient clinic of the Department of Periodontics, Maulana Azad Institute of Dental Sciences, New Delhi, were screened. A single examiner recorded probing depth, clinical attachment loss, and bleeding on probing, and recruited subjects based on the inclusion and exclusion criteria. A computer-generated randomization table was used to assign subjects into two groups: Group I: 0.2% CHX and Group II: 0.1% stabilized ClO₂ (Figures 1, 2).

Two phases of the study

Phase I (Sample Collection)

**Figure 3:**
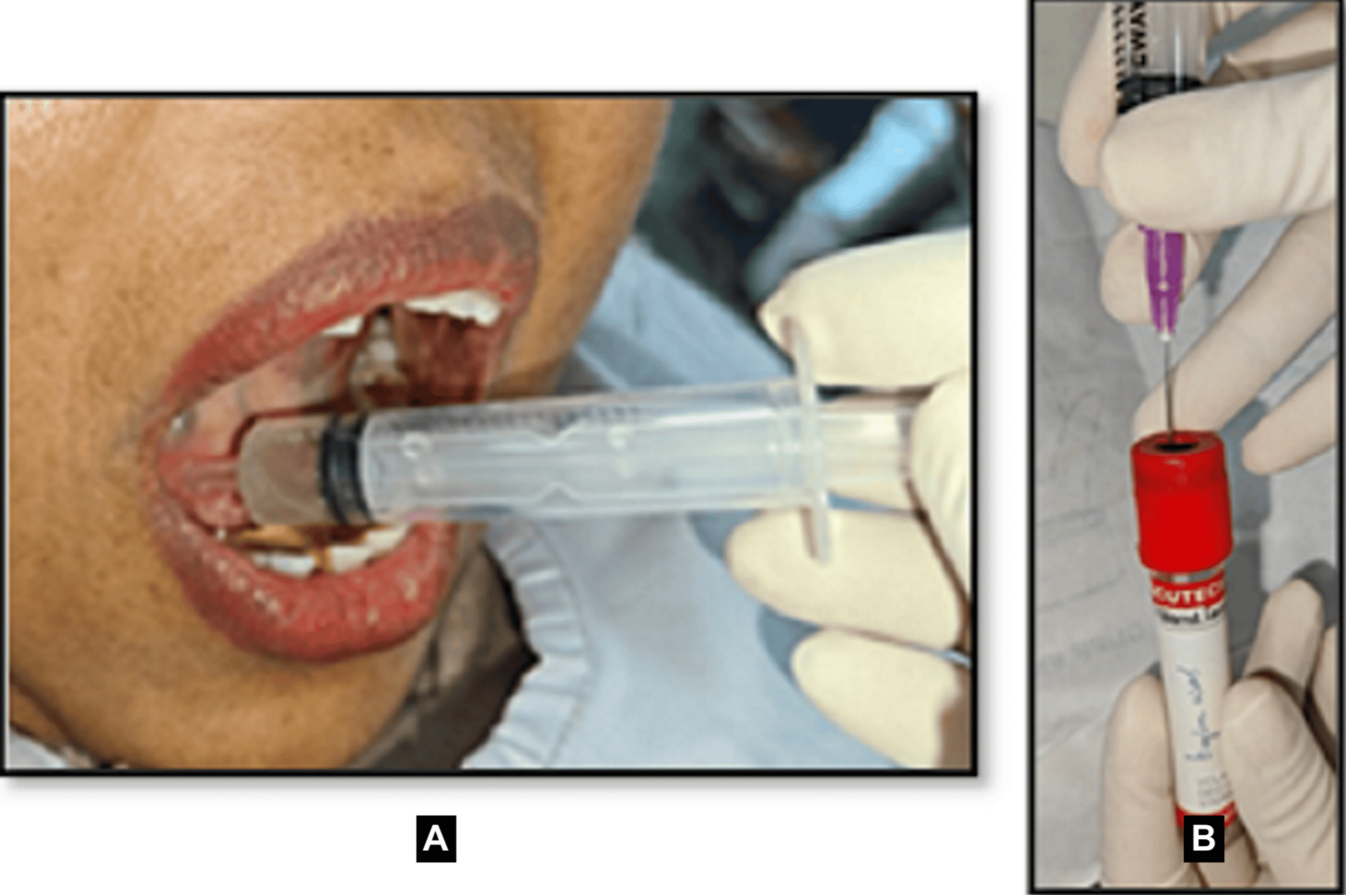
(A) Collection of the saliva sample from the floor of the mouth with the help of a syringe. (B) Transferring the saliva sample into a plain Vacutainer vial

Subjects in both groups were instructed not to swallow or spit saliva during sample collection. Unstimulated saliva that naturally pooled in the floor of the mouth was collected using a sterile syringe and transferred into a fresh plain vacutainer vial. These samples were labeled “Before Wash” (BW) and assigned a unique subject-specific code (Figures 3A, 3B).

Subsequently, subjects in Group I were given 10 mL of 0.2% CHX and instructed to perform a pre-procedural rinse by swishing the solution in the oral cavity for 60 seconds, after which they were asked to expectorate. Subjects in Group II were given 10 mL of 0.1% stabilized ClO₂ and instructed to follow the same procedure. Mouthwash for rinsing was poured into the disposable cup just prior to rinsing to limit contamination. All rinses were performed under supervision. After five minutes, subjects were instructed to allow saliva to pool again in the floor of the mouth. A post-rinse saliva sample (1-2 mL) was then collected using a sterile syringe and transferred into a fresh plain Vacutainer vial labeled as “After Wash” (AW), followed by the subject’s unique code.

In addition to microbial assessment, participants were asked to rate the taste and odor of the assigned mouthwash using a Visual Analog Scale (VAS), where a score of 10 indicated the most pleasant experience and 0 indicated the least pleasant. Plain water was used as the reference standard and assigned a score of 10. This subjective evaluation provided insight into the palatability and overall acceptability of each mouthwash, both of which are important factors influencing patient compliance in routine dental care.

Phase II (Sample Culture/Microbial Analysis)

The collected samples were transported in plain Vacutainer vials to the Department of Microbiology at Maulana Azad Medical College, New Delhi, for culture. A single trained laboratory personnel processed the samples within two hours of collection.

Using a sterile pipette, 100 µL of saliva (the standard inoculum volume for the lawn culture technique [16]) from the “BW” sample was aspirated and inoculated onto blood agar and MacConkey’s agar using the lawn culture technique. The plates were incubated aerobically at 37°C for 24 hours.

An additional 100 µL from the same “BW” sample was cultured in a similar manner and incubated anaerobically at 37°C for 48 hours. Anaerobic Gas Pak jars were used.

Similarly, 100 µL of saliva from the “AW” sample was inoculated onto blood agar and MacConkey’s agar using the lawn culture technique and incubated aerobically at 37°C for 24 hours.

Another 100 µL from the same sample was cultured under anaerobic conditions and incubated at 37°C for 48 hours.

**Figure 4:**
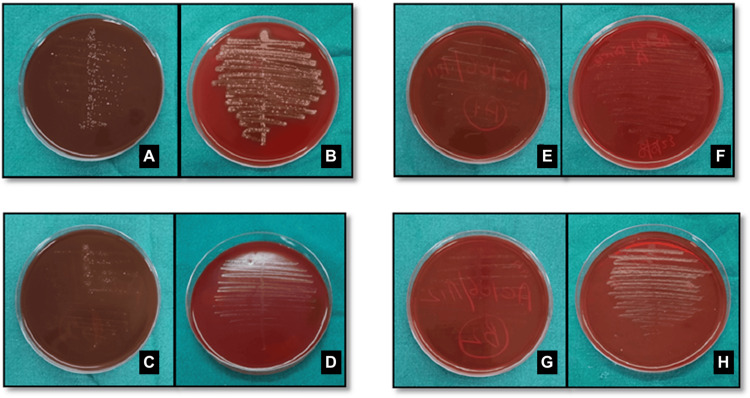
(A,B) Aerobic culture on blood agar plate before wash. (C,D) Aerobic culture on blood agar plate after wash. (E,F) Aerobic culture on MacConkey’s agar plate before wash. (G,H) Aerobic culture on MacConkey’s agar plate after wash

**Figure 5:**
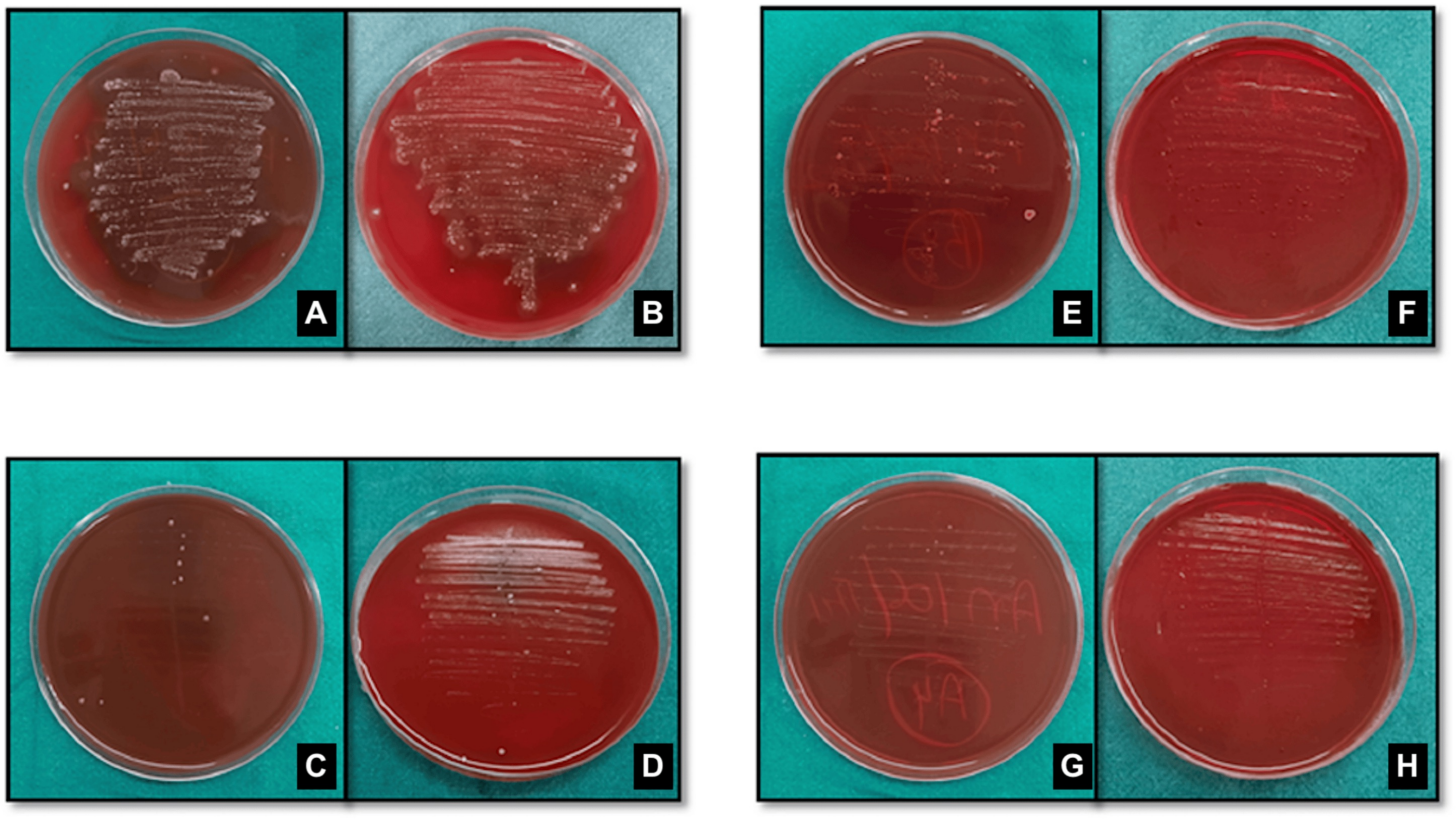
(A,B) Anaerobic culture on blood agar plate before wash. (C,D) Anaerobic culture on blood agar plate after wash. (E,F) Anaerobic culture on MacConkey’s agar plate before wash. (G,H) Anaerobic culture on MacConkey’s agar plate after wash

After completion of the incubation periods (24 hours for aerobic and 48 hours for anaerobic cultures), microbial growth was quantified and expressed in logarithmic form as colony-forming units per milliliter (CFU/mL) (Figures 4A-H, Figures 5A-H).

## Results

**Figure 6:**
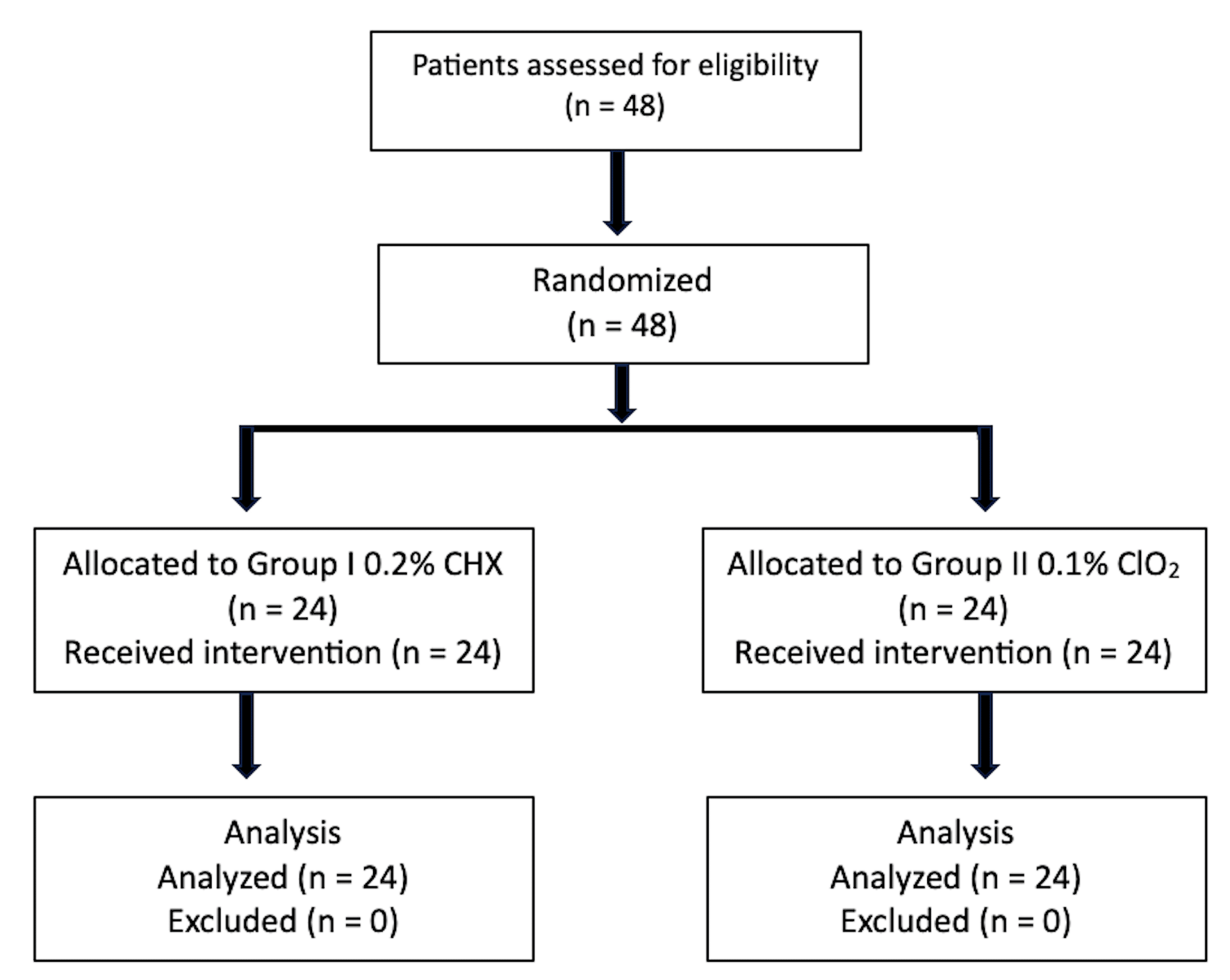
CONSORT flowchart showing participant follow-through in the study CONSORT: Consolidated Standards of Reporting Trials Flowchart created using Microsoft Word (Microsoft Corporation, Redmond, Washington).

A total of 48 patients were randomized equally to the CHX and ClO₂ groups, and all participants completed the study and were included in the analysis (Figure 6). This study evaluated and compared the antimicrobial effectiveness of two different pre-procedural mouthrinses by assessing aerobic and anaerobic bacterial loads in saliva samples, measured as CFUs × 10^6^. Statistical analysis was performed using paired t-tests to compare bacterial counts within groups at baseline and after rinsing. A p-value ≤ 0.05 was considered statistically significant.

Intragroup comparison of aerobic bacterial load

Only Group 1 exhibited a statistically significant decrease in aerobic bacterial count following the mouthrinse (p = 0.00), as shown in Table 1.

**Table 1:** Intragroup comparison of aerobic bacterial count (10*6) among both the groups at different time intervals using a paired t-test * p≤0.05 is considered statistically significant.

Group	Time	Mean±SD (10^6^)	t-value	p-alue
Group 1	Before Wash	16.74±27.46	3.06	0.0*
	After Wash	3.02±6.75		
Group 2	Before Wash	9.24±14.03	1.07	0.18
	After Wash	5.46±14.21		

Intragroup comparison of anaerobic bacterial load

Within-group analysis showed a statistically significant reduction in anaerobic bacterial load in both groups (Group 1: p = 0.03; Group 2: p = 0.00). These findings indicate that both rinses were effective in reducing anaerobic bacterial counts from baseline (Table 2).

**Table 2:** Intragroup comparison of anaerobic bacterial count (10*6) among both the groups at different time intervals using a paired t-test * p≤0.05 is considered statistically significant.

Group	Time	Mean±SD (10^6^)	t-value	p-value
Group 1	Before Wash	33.12±64.96	2.19	0.03*
	After Wash	4.62±10.40		
Group 2	Before Wash	9.43±13.03	3.09	0.00*
	After Wash	1.91±4.31		

For intergroup comparisons of both aerobic and anaerobic bacterial loads, statistical analysis was performed using independent t-tests. A p-value ≤ 0.05 was considered statistically significant.

Intergroup comparison of aerobic bacterial load

The mean post-rinse aerobic bacterial counts did not differ significantly between the two groups (p = 0.45), as shown in Table 3.

**Table 3:** Intergroup comparison of aerobic bacterial count (10*6) after wash using an independent t-test

Group	Mean±SD (10^6^)	t-value	P-value
Group 1	3.02±6.75	-0.76	0.45
Group 2	5.46±14.21		

Intergroup comparison of anaerobic bacterial load

Although both groups demonstrated a reduction in bacterial count after rinsing, the difference between the groups was not statistically significant (p = 0.24) (Table 4).

**Table 4:** Intergroup comparison of anaerobic bacterial count (10*6) after wash using an independent t-test

Group	Mean±SD (10^6^)	t-value	p-value
Group 1	4.62±10.40	1.17	0.24
Group 2	1.91±4.31		

VAS scores were analyzed using the Mann-Whitney U test due to their ordinal nature (Table 5). Group 2 demonstrated a significantly higher mean rank (34.21) compared to Group 1 (14.79) (Z = −4.88, p = 0.00), indicating that participants found the mouthrinse used in Group 2 to be significantly more pleasant.

**Table 5:** Intergroup comparison of VAS scores after usage among both groups using the Mann-Whitney U test * p≤0.05 is considered statistically significant. VAS: Visual Analog Scale

Group	Mean rank	Z-value	p-value
Group 1	14.79	-4.88	0.00*
Group 2	34.21		

Based on the data and statistical analysis, both Group 1 and Group 2 showed a significant reduction in anaerobic bacterial counts following pre-procedural rinsing, whereas only Group 1 demonstrated a statistically significant reduction in aerobic bacterial count.

Intergroup comparisons of post-rinse bacterial counts revealed no statistically significant differences for either aerobic or anaerobic bacteria, indicating that both mouthrinses were comparably effective in reducing microbial load.

Additionally, Group 2 received significantly higher VAS scores for taste and odor, indicating better patient acceptability and palatability of the mouthrinse used in that group.

## Discussion

Dental treatments, particularly those involving ultrasonic instruments, are known to generate aerosols that can carry high levels of microbial contaminants. These aerosols pose a substantial risk of cross-infection for both dental professionals and patients. To minimize this risk, the use of a pre-procedural mouthrinse has become a widely recommended practice. It is a simple, quick, and low-cost intervention that can significantly reduce the number of bacteria in the oral cavity and, consequently, in aerosols generated during treatment.

The effectiveness of such rinses has been well documented. For instance, Fine et al. reported that using an antiseptic rinse prior to treatment resulted in a 94% reduction in viable bacteria in aerosols compared with patients who did not rinse [17]. A systematic review by Nagraj et al. further supported these findings, demonstrating that mouthrinses containing CHX, CPC, or essential oils can reduce microbial aerosols by up to 78.9% [18]. Although the 2022 Cochrane Review highlighted some inconsistency in study quality, it still supported the role of pre-procedural rinsing in infection control within dental settings [18].

Among the available mouthrinses, CHX has long been considered the “gold standard” due to its broad-spectrum antimicrobial activity and substantivity. Its mechanism of action involves disruption of the bacterial cell membrane and interference with intracellular components [19]. De Albuquerque et al. reported that a 0.12% CHX rinse reduced *Staphylococcus aureus* and *Streptococcus mutans* levels by more than 99%, outperforming CPC [20]. Similarly, Veksler et al. observed that CHX reduced aerobic bacterial levels by 77% at 30 minutes and 96% at 60 minutes post-rinse [21]. Retamal-Valdes et al. also confirmed the effectiveness of CHX in reducing bacterial aerosols, with comparable results reported for CPC-based rinses containing zinc and fluoride [15].

In recent years, stabilized ClO₂ has emerged as a promising alternative. It exhibits broad antimicrobial activity and offers additional advantages, such as improved taste, absence of staining, and greater patient acceptability. ClO₂ acts by selectively oxidizing bacterial proteins and disrupting essential cellular functions. In laboratory studies, Lundstrom et al. demonstrated that ClO₂ effectively eliminated key anaerobic bacteria, including *Fusobacterium nucleatum* and *Peptostreptococcus micros*, which are commonly associated with periodontal infections [22]. Another in vitro study reported that ClO₂ achieved 100% kill rates against several oral pathogens within five minutes, in some cases acting faster than CHX [23].

Beyond its antibacterial effects, ClO₂ has also shown antiviral potential. Travis et al. demonstrated that mouthrinses containing ClO₂ could significantly reduce viral load, including SARS-CoV-2 [24,25].

In the present study, both CHX and ClO₂ demonstrated a significant reduction in anaerobic bacterial counts in patients with periodontitis following a single pre-procedural rinse. However, only CHX produced a statistically significant reduction in aerobic bacteria. Although ClO₂ also reduced aerobic CFUs, the reduction did not reach statistical significance. Intergroup comparisons revealed no significant differences in overall antimicrobial efficacy between the two rinses, suggesting that both are effective in reducing microbial load prior to dental procedures.

Importantly, participants in the ClO₂ group reported significantly higher satisfaction scores for taste and odor, which may enhance patient compliance in routine clinical practice.

The following recommendations may be considered to further reduce the risk of cross-contamination in dental settings: (1) pre-procedural mouthrinsing should be considered a routine step prior to all dental procedures, especially those that generate aerosols; (2) early detection of periodontal disease should be promoted through regular screening programs; (3) patients should be educated about the association between periodontal disease and systemic health conditions; and (4) patients should be motivated to maintain good oral hygiene and to discontinue harmful habits such as tobacco use.

Further studies involving a larger number of participants and a wide range of oral conditions and dental procedures are needed to better understand the effectiveness of pre-procedural mouthrinses. Such research would help clarify their role in reducing microbial load and lowering the risk of cross-contamination in routine dental practice.

## Conclusions

Within the limitations of the present study, it can be concluded that CHX remains a reliable and highly effective antimicrobial mouthrinse, particularly in reducing both aerobic and anaerobic bacterial loads. However, stabilized ClO₂ demonstrates comparable antimicrobial efficacy, especially against anaerobic organisms, while offering advantages such as better taste, absence of staining, and improved patient acceptability. In cases where CHX is contraindicated or poorly tolerated, ClO₂ may serve as a practical and patient-friendly alternative for infection control in dental practice.
